# Sex differences in behavioral, cognitive and voluntary ethanol-intake effects in Dexamethasone-induced depression-like state in Wistar rat

**DOI:** 10.3934/Neuroscience.2022012

**Published:** 2022-04-29

**Authors:** Laaziz Abderrahim, El Mostafi Hicham, Elhessni Aboubaker, Azeroil Fatima, Touil Tarik, Boumlah Soufiane, Mesfioui Abdelhalim

**Affiliations:** 1 Biology And Health Laboratory, Faculty of Sciences, Ibn Tofail University, Kenitra, Morocco; 2 Higher Institute of Nursing and Health Professions of Rabat, Morocco

**Keywords:** sexual dimorphism, anxiety-depressive like behavior, dexamethasone, voluntary ethanol intake, cognitive impairment

## Abstract

The stress response is attached to psychosomatic and psychiatric disorders. Therefore, it is important to comprehend the underlying mechanisms influencing this relationship. Moreover, men and women respond differently to stress–both psychologically and biologically. These differences should be studied to have an enhanced understanding of the gender difference. However, researches shedding light on sex dimorphism implication have historically been insufficient. Based on observations that advocate the inclusion of sex as a biological variable in stress response, the present study was designed to explore sex differences in (i) depressive-like, (ii) anxiety-like behaviors, (iii) cognitive-like performances, and (iv) voluntary ethanol intake (VEI) in Wistar rat submitted to dexamethasone (DEX)-stress simulation. Rats were administered daily with DEX (1.5 mg/kg, s.c., 21 days) or vehicle. Behavior, cognitive, and VEI states of rats were evaluated in the following paradigms: forced swimming test (FST); saccharin preference test (SPT); open field test (OFT); elevated plus-maze test (EPMT); novelty suppressed feeding test (NSFT); spatial learning and memory in Morris water maze test (MWMT); VEI in two-bottle choice paradigm. DEX-treated rats showed a set of depression-like behaviors: increased time of immobility; reduced preference for saccharin consumption; increased anxiety-like behavior; cognitive impairments; and enhanced VEI. Sexual dimorphism was recorded in this study. Females were more impaired in FST, SPT, EPMT, NSFT, and VEI. Results demonstrate that DEX-treatment induced a behavioral alterations related to anxiety-depressive-like state with learning and memory impairments; confirm the facilitatory role of glucocorticoids on VEI and reveal sexual dimorphism in stress response.

## 1. Introduction

Chronic stress can lead to a variety of psychological alterations, including anxiety-depression and alcohol intake. In a great body of epidemiological surveys, stress-induced psychopathologies co-occur as comorbidities at high rates [1]. Stressors like threatening situations trigger serial neuroendocrine events orchestrated by systems of stress with the hypothalamic-pituitary-adrenal axis (HPA) considered as the main actor. Activation of the HPA axis initiates secretion of hypothalamic corticotropin-releasing hormone, which later led to pituitary adrenocorticotropin releasing hormone liberation, resulting in adrenal glucocorticoids secretion into the blood circulatory system [2]. Glucocorticoids then act on target tissues, causing physiological changes that prepare the body for stress and then establish a state of pre-stress functioning. In normal cases, HPA axis involvement is primordial for surviving because it intervenes to conserve basic homeostasis [3]. However, prolonged HPA axis activation may result in negative physiological effects and conduct a deep influence on brain function [3]–[5]. For example, elevated glucocorticoids exposure induces a diminution of hippocampal glucocorticoid receptors that weakens hippocampus capacity to regulate negative glucocorticoid feedback [6]. Altered feedback causes more hypersecretion of glucocorticoids, which led to neuronal changes in many brain structures, encompassing the hippocampus [7] and amygdala [8]. Persistently elevated glucocorticoids within the hippocampus results in dendritic remodeling of CA3 neurons, impaired neurogenesis, and even leads to cell death [4],[5],[9].

Recurrent stress and hyperactivation of the HPA axis have been encountered with depression occurrence [10]. More than half of depressed patients exhibit elevated circulating cortisol [11]. Interestingly, patients with depression also declare cognitive impairments and exhibit hippocampal volume reductions [12]. Also, individuals suffering from Cushing's problem, which is characterized by persistently elevated cortisol levels, manifest abnormally high depression incidences [13]. The depression severity is often exacerbated by co-occurring anxiety symptoms. Effectively; cases, when depression occurs independently from any anxiety symptoms, are very rare [14].

Hyper circulating glucocorticoid is a common feature observed in the psychopathology of depression and alcohol use disorders (AUDs). A large body of preclinical and clinical studies shows that stressors can enhance motivation for alcohol consumption in those with or without AUDs [15]. Reward and stress systems interactions constitute the neurobiological ground underlying stress-induced elevations in alcohol intake. Voluntary alcohol intake is facilitated by glucocorticoids, this was concluded from results of adrenalectomy which provoked diminished alcohol intake in Wistar and alcohol-preferring rats [16], while an intracerebro-ventricular infusion of glucocorticoids enhances voluntary alcohol intake in Wistar rats [17]. Even more, corticosterone (Cort) itself is self-administered in plasma levels comparable to that caused by stress [18]. These facilitatory roles of glucocorticoids on alcohol intake and their reinforcing properties are very mediated by glucocorticoid receptors expressed in the reward system. Drugs share a common property to increase dopamine secretion in the Nucleus Accumbens [19]. This property leads to raising the question of glucocorticoid-receptor-activation can increase dopaminergic activity. Data scrutinizing this question have demonstrated that elevated glucocorticoid levels induced by stress influence mesolimbic dopaminergic activity, led by several mechanisms in big dopamine release, and finally possible facilitation of drug-induced reinforcement [20].

Stress and stress-related complications have largely been studied on many levels with different approaches. However, preclinical and human research that shed light on the implication of sex dimorphism has historically been insufficient. For example, an investigation of sex bias in research declared male bias in many fields, with neuroscience exhibiting the marked bias, including those related to stress responses [21]. This female underrepresentation has been explained by some reasons, one essential is that studying female rodents has been judged challenging for their short estrous cycle and because of ovarian hormones that can induce neuronal plasticity [22]. In addiction studies, males are included at elevated rates than females; also researches including both sexes do not examine results in a sex-dependent way. Gender is an essential determinant in human health concerns. The tendency for the sex-specific prevalence rates in many mental and physical disorders has been apparently reported. In fact, sex differences appear at numerous levels of body organization including brain anatomy and connectivity [23]–[26]. In an attempt to surpass male bias, some recommendations have been lined, for example, the National Institutes of Health demanded females inclusion in research (1993) [27] and, more recently, sex as a biological variable (SABV) in basic and preclinical studies [28]. The SABV inclusion is an important point to improve rigor and reproducibility in scientific results, which guide clinical research and practice [28].

Based on the above-mentioned observations and under recommendations which advocate the inclusion of sex as a biological variable in the stress response, the present study was designed to explore the sex differences in (i) depressive-like, (ii) anxiety-like behaviors, (iii) cognitive-like performances and (iv) voluntary ethanol intake (VEI) in Wistar adolescent rats submitted to chronic DEX-stress simulation. By this work, we wanted to focus on an important issue which is sex differences in the context of neuropsychiatry. It is crucial to shed light on sex discrepancies between males and females. Indeed, stress seems to affect more females remains unclear how and at what level. Considering sex-specificities may provide critical clues for the design of preventive strategies and therapeutic interventions. Synthetic glucocorticoids are frequently used in medicine for their anti-inflammatory and immunosuppressive actions [29]. DEX used in this study as synthetic glucocorticoid is more potent than Cort; it has a long half-life, higher glucocorticoid activity, and no mineralocorticoid activity [30]. Also; DEX, by acting selectively on glucocorticoid receptors (GR), provides an experimental opportunity to study the direct effect of prolonged GR activation on depression induction. Many studies have reported that DEX chronic administration induced behavioral changes related to a depressive-like disorder such as despair, anhedonia, and weight loss [29],[31]; anxiety-like state [31]; structural changes such as remodeling of microglia and neuronal morphology [31]. Skupio et al. [32] have shown that 21 days of DEX treatment in mice induces depressive-like symptoms, decreases expression of glucocorticoid receptor gene, increases expression of Fkbp5 and Sgk1 genes, and decreases expression of astroglial marker GLAST. Even acute use of DEX at prenatal has been reported to induce long-lasting behavioral changes; Ferreira et al. [33] showed that exposure to stress mediators in critical periods of development negatively affects behavior and metabolism. Many studies related to DEX stress simulation have been realized in rats or mice at adult age [29],[32],[34],[60]. Others focused on the long-lasting effects of prenatal corticoid treatment [31],[33]. This study has used adolescent rats. Adolescence is a critical window of brain development; during this stage, the brain is sensitive to stress reactivity, which heightens vulnerability to psychiatric disorders later in life [35],[36].

## 2. Materials and methods

### 2.1. Animals and drugs

**Figure 1.**
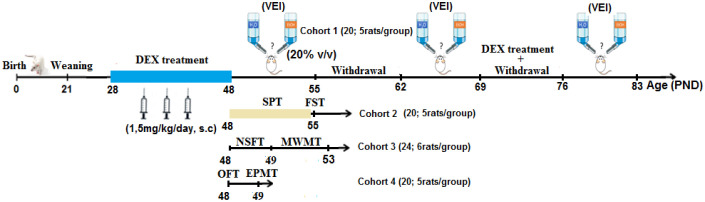
Time-line representing experimental design, In this study, we aimed to assess sex differences in behavioral, cognitive, and voluntary ethanol consumption effects induced by a stress simulation represented by DEX treatment in rats aged 28 ± 3.5 postnatal days (PND). Wistar rats were administered daily with DEX (1.5 mg/kg, s.c, 21 days) or vehicle. The behavior and cognitive states of rats were evaluated in these paradigms: forced swimming test (FST); saccharin preference test (SPT); open field test (OFT); elevated plus-maze test (EPMT); novelty suppressed feeding test (NSFT); spatial learning and memory in Morris water maze test (MWMT); voluntary ethanol intake (VEI) in two-bottle choice paradigm.

The study was conducted on 84 adolescent Wistar rats both sex weighting initially 80.75 ± 1.76 g and aged 28 ± 3.5 postnatal days. Animals were lived under a natural diurnal cycle and 23 ± 1 °C temperature. Food and water were given ad libitum. Animals were divided into two main groups (n = (21 males + 21 females)/group). One group received a daily single sub-cutaneous injection (s.c.) of DEX at a dose (1.5 mg/kg) for 21 consecutive days, while the other group was administrated for the same duration with the vehicle. After 21 days of injection, rats underwent behavioral and cognitive tests in different cohorts. The experimental procedures are summarized in Figure 1. The concentration of DEX injections was an adaptation from the study of [29]. DEX injections were realized daily between 9:00 a.m. and 10 a.m. The whole work of this study has been accomplished in Biology and Health Laboratory at Ibn Tofail University (Kenitra, Morocco). All experimental manipulations and testing have been realized in compliance with the NIH Guide for the Care and Use of Laboratory Animals. Animals were weighed every week throughout the experiment.

### 2.2. Depressive-like behavior measurement

#### 2.2.1. Forced swim test

The forced swim test (FST) was used to assess despair-based depressive-like behaviors. The procedure of this test was adapted from that described by Porsolt [37]. Rats were singly emerged in a glass cylinder heightened 50 cm with 30 cm diameter. The apparatus contained 27 cm of water without any escape exits. The water was warmed to at 26 ± 1 °C, and it was changed for each rat. During each 6 minutes, trial rat was quietly emerged into the water then removed and let dry with lignin in a separate cage before returning to the home cage. Time immobility was recorded visually uniquely for the 4 last minutes of the test. Immobility is determined as the absence of escape-oriented movements such as swimming, diving, rearing, and jumping but not those necessary to keep head above the water [38].

#### 2.2.2. Sucrose preference test

The Sucrose preference test (SPT) was used to assess anhedonia as another side of depressive-like behaviors. This test is based on saccharin solution intake measurement. Rats individually housed were allowed to drink in a free-choice manner from two bottles for one week. One with tap water while the other contained saccharin solution (2%). Bottles position was changed every 12 hours per day to avoid eventual place preference. In this version of SPT, there was no food and water privation. Evaluation of daily liquid consumption was given by weighing the bottles. The saccharin preference was calculated uniquely for the 4 last days as saccharin percentage compared to total liquid consumed.

### 2.3. Anxiety-like behaviors and locomotor activity measurement

#### 2.3.1. Open field test (OFT)

The procedure adopted in this study was similar to that previously reported [39]. The device was a square wooden made (100 cm × 100 cm) enclosed with 40 m high walls. The apparatus was divided into 25 squares (16 peripherals, 9 centrals) and placed under illumination (100 w, 2 m above) during behavioral testing. The test duration has been determined in 10min; each animal was placed in the center then video-recorded. After each trial, the apparatus was cleaned with 70% ethanol. Anxiety-like behavior was estimated from time spent in central squares and the locomotor activity from the total distance traveled during 10 min testing.

#### 2.3.2. Elevated plus maze test (EPMT)

The procedure is considered to be the main model of anxiety in animals. It is founded on a conflict creation between exploratory behavior and fear from exposed parts of the apparatus [40]. The EPM utilized was a wooden plus-shaped device 70 cm elevated from the floor. The two facing arms (50 cm × 10 cm) are limited by three 40 cm high walls, while the others were bordered by 0.5 cm edge to avoid animal fall. The intersection of four arms determined a central area (10 cm × 10 cm). The apparatus was illuminated by 100 w placed above the central area. Rats were placed in the central area facing an open arm for 5 min then video-recorded. The device was cleaned after each trial with 70% ethanol.

#### 2.3.3. Novelty suppressed feeding test (NSFT)

The test is based on the creation of conflict in animals between the desire to eat and the fear of open and exposed areas. Animals in front of this conflict take a coping time that reflects anxiety-like behavior degree [41]. Before testing, rats were 24 h food-private; then each rat was placed in the corner of the open-field arena. Food was placed in the center on a circular white paper; the floor was covered with sawdust. The latency to begin eating was recorded. Rats were allowed 10 min to stay tested if they failed to go eat. The sawdust was mixed after each rat session to avoid olfactory perturbation.

### 2.4. Spatial learning and memory

The Morris water maze test (MWMT) used in this study is as previously described [42]. The device was a circular pool (160 cm diameter, 40 cm height) divided into four quadrants by North, South, East, and west direction given to four points arbitrarily chosen. Water was mixed with milk powder to warrant water opacity. In one quadrant center, there was a transparent platform (10 com diameter, 2 cm immersion) serving as an escape used by rats during the training session. The training session was carried out in 5 days, the first day was considered as pre-training day; animals were immerged three times for 20 s as a water habituation. After, the rats were trained to find the invisible immerged platform using extra-labyrinth visual information. The training was based on four swims per day separated by 10–15 min. One swim consisted in putting the rat in one arbitrarily direction point facing the wall pool and allowing 120 s to reach the platform. Each rat was let 10 s in the platform to memorize its location. Learning in this training period was considered as the time to reach the invisible platform. When the rat failed to find the platform during 120 s it was then gently oriented to the platform. One hour, after the last session, the platform was removed then rats were allowed to swim for 120 s. Time spent in the quadrant where the platform was during the training period was recorded [43].

### 2.5. VEI in two bottle choice paradigm

After the 21 of DEX injection, rats in an isolated cohort (n = 6 rats/group) have free choice between two bottles, one filled with ethanol (20% V/V) while the other contained tap water. Ethanol liquid was diluted in tap water from ethanol 96°. To avoid position preference bottles were daily interchanged. Ethanol and water liquid intake was daily recorded by measuring the weight of bottles; then the ethanol intake was expressed as (g/kg/day). This amount was calculated uniquely for the four last days of each week of measurement. Daily voluntary alcohol intake was carried out over three recording sessions. Each session lasted one week. The first session came right after the last injection of DEX, the second started after a week of withdrawal, and the third session was performed after a week of withdrawal with additional daily injections of DEX (see experimental schedule for details, Figure 1). The VEI measurement was adapted from the published work of our laboratory with ELmostafi et al. [44] and Peñasco et al. [45].

### 2.6. Statistical methods

Except OFT and EPMT which were video-recorded then analyzed by video tracking Panlab smart setup version 3.0, the other behavioral tests were visually analyzed. The study data were analyzed by Graph pad prism version 8, and they were expressed as mean ± standard error. Comparison between groups was statistically given by a two-way ANOVA test. A three-way ANOVA was carried out for the body weight evolution and the learning period in MWMT. All post-hoc comparisons were given by Tukey test. *p*< 0.05 was considered a significant difference.

## 3. Results

### 3.1. Body weight

**Figure 2.**
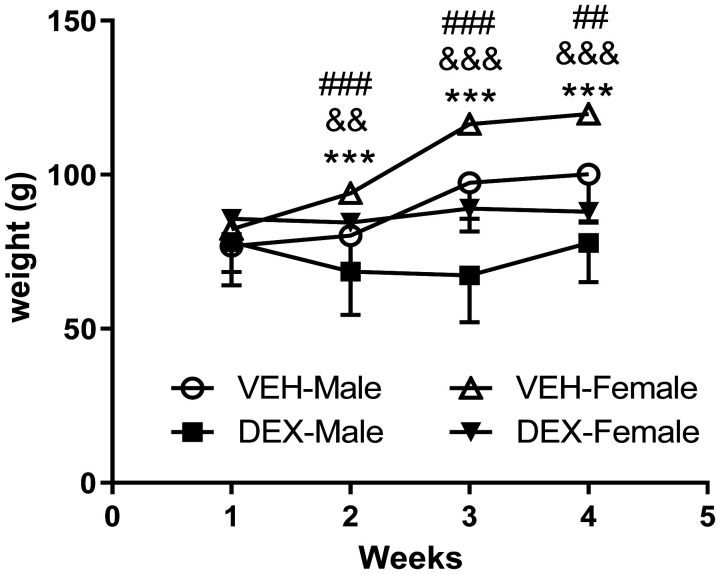
Effect of repeated dexamethasone administration on rat body weight. DEX (1.5 mg/kg) was daily and subcutaneously administered for 21 days, starting at 28 days of life. A significant reduction in body weight was observed after one week of administration. DEX Dexamethasone, VEH vehicle. ** *p* < 0.01, *** *p* < 0.001 significant difference between vehicle and dexamethasone male groups; && *p* < 0.01, &&& *p* < 0.001 significant difference between vehicle and dexamethasone female groups; ## *p* < 0.01, ### *p* < 0.001 significant difference between male and female dexamethasone groups. Data represent means ± SEMs (n = 7 rats/group).

Figure 2 shows the evolution, throughout the 21 days of treatment, of mean body weight of rats. After the first week of DEX administration body weight of treated rats was significantly reduced. A three-way ANOVA for repeated measures analysis indicated significant effects of time (F (3, 60) = 76.10; *p* < 0.001), treatment (F (1, 20) = 13.25; *p* < 0.01), sex (F (1, 20) = 16.91; *p* < 0.001), an interaction between time and treatment (F (3, 60) = 10,22; *p* < 0.001) and between time and sex (F (3, 60) = 67.58; *p* < 0.001). Post-hoc Tukey's multiple comparisons test revealed more reduction of body weight in male DEX-treated rats compared to other groups.

### 3.2. Depressive-like behaviors: despair and anhedonia

**Figure 3.**
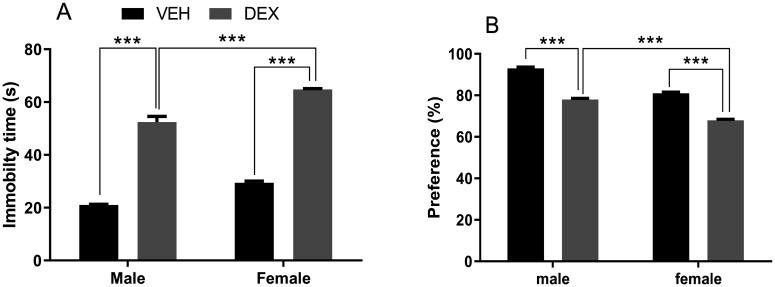
Effect of repeated dexamethasone administration on behavioral despair as assessed in the forced swimming test (A) and anhedonia as evaluated by the saccharin preference test (B). DEX treatment resulted in elevated immobility time, the treated females showed more immobility time (A); and reduced saccharin preference, the treated females showed the smallest saccharine preference (B). Data represent means ± SEMs (FST n = 5 rats/group; SPT n = 5 rats/group). VEH, vehicle group; DEX, dexamethasone group. *** *p* < 0.001.

To assess if prolonged DEX administration (1.5 mg/kg, s.c.) can induce depressive-like behavior in rats, an FST was used. Figure 3A revealed that's DEX treatment conducted to more immobility time as compared with the non-treated group, which indicates that DEX injections have depressive-like behavior. Two-way ANOVA exhibited significant effects of treatment (F (1, 16) = 831.6; *p* < 0.0001) and sex (F (1, 16) = 80.37; *p* < 0.0001). Post-hoc Tukey's multiple comparisons test revealed that female treated rats exhibited greater immobility time compared to treated males (*p* < 0.001).

On other hand, anhedonia represents one of the main core symptoms related to depressive-like behavior. An SPT was used, as a way to evaluate anhedonia in rats, after the 21 DEX treatment days and before FST. Figure 3B showed that DEX treatment resulted in reduced saccharine intake compared to the non-treated rats. Analysis by Two-way ANOVA indicated significant impacts of treatment (F (1, 12) = 574.0; *p* < 0.0001) and sex (F (1, 12) = 354.6; *p* < 0.0001). Post-hoc Tukey's multiple comparisons test revealed that female treated rats showed more reduction in saccharine consumption compared with male treated rats (*p* < 0.001).

### 3.3. Anxiety-like behavior and locomotor activity

**Figure 4.**
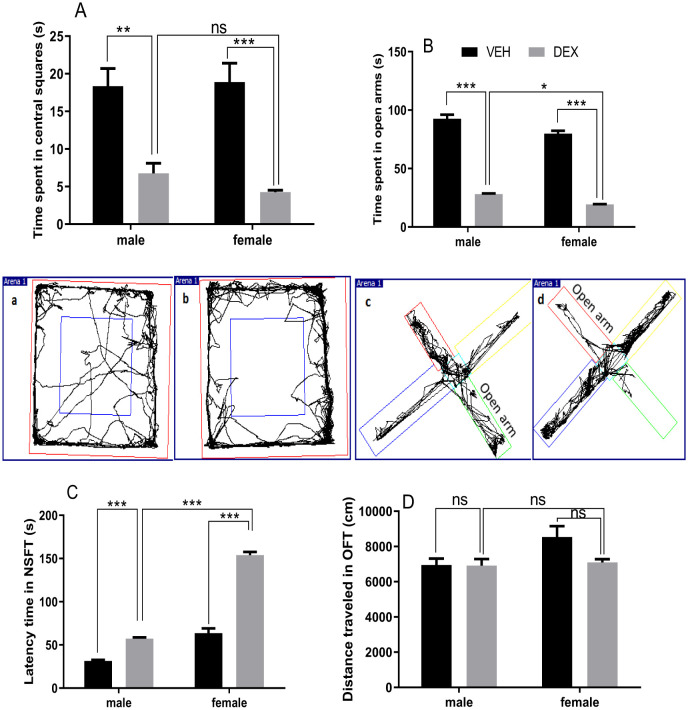
Effect of repeated dexamethasone administration on time spent in central squares of OF arena (A; a and b examples of the illustration given by video tracking panlab smart setup respectively of control and treated rats), time spent in open arms of EPMT (B; c and d examples of the illustration given by video tracking panlab smart setup respectively of control and treated rats), latency time of NSFT (C) and total distance traveled in OF arena (D). DEX treatment resulted in a reduced time spent in central squares (A), a reduced time spent in open arms of EPMT with treated females showed the shorter time in (B), a greater latency time with treated female exhibited the highest time to begin eating (C) and no effect on ambulatory locomotor activity assessed as distance traveled in OF arena (D). Data represent means ± SEMs (OFT n = 5 rats/group; NSFT n = 6 rats/group; EPMT n = 5 rats/group). VEH, vehicle group; DEX, dexamethasone group. ns non-significant, * *p* < 0.05, ** *p* < 0.01, *** *p* < 0.001.

Evaluation of anxiety-like behaviors has been realized by three test indicators: time spent in central squares of the OFT arena, time passed in open arms of EPMT, and latency time of NSFT. As shown in Figure 4A, DEX administration conducted to a decreased time spent in central squares, Analysis by Two-way ANOVA indicated significant impacts of treatment (F (1, 16) = 50.05; *p* < 0.0001). In Figure 4B, DEX administration resulted in diminished open arms time. Analysis by Two-way ANOVA indicated significant impacts of treatment (F (1, 16) = 839.1; *p* < 0.0001) and sex (F (1, 16) = 25.05; *p* < 0.001). Post-hoc Tukey's multiple comparisons test revealed that female treated rats showed decreased time compared with male treated rats (*p* < 0.05). In Figure 4C, latency time before going to eat was significantly increased in DEX-treated rats. Analysis by Two-way ANOVA indicated significant impacts of treatment (F (1, 20) = 267.08; *p* < 0.0001), and sex (F (1, 20) = 328.44; *p* < 0.0001). Post-hoc Tukey's multiple comparisons test revealed that female treated rats exhibited more latency time compared with male treated rats (*p* < 0.001). To investigate the DEX injection effect on locomotor activity, a measurement of ambulatory distance was evaluated in OF arena. Figure 4D indicates that prolonged DEX treatment did not influence this locomotor activity parameter.

### 3.4. Spatial learning and memory in MWMT

**Figure 5.**
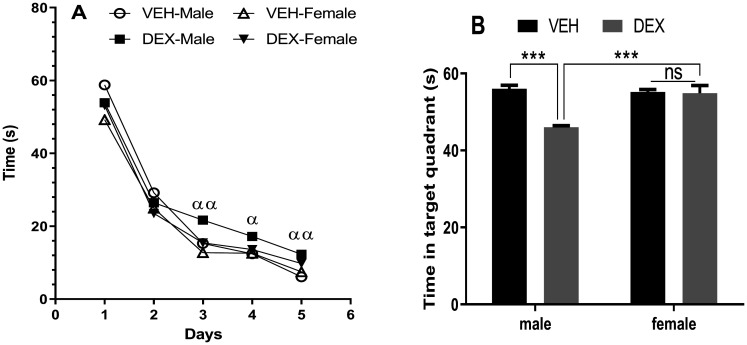
Effect of repeated dexamethasone administration on spatial learning performance and memory. (A) Latency to find the submerged platform during the five days of training, (B) time spent in the target quadrant without platform during the probe test. Chronic DEX administration affected only male rats and resulted in a greater latency to find the submerged platform (A), and a low time spent in the target quadrant (B). Data represent means ± SEMs (n = 6 rats/group). VEH vehicle, DEX dexamethasone. α *p* < 0.05, αα *p* < 0.01 significant difference between vehicle and dexamethasone male groups; *** *p* < 0.001, ns non-significant.

In MWMT, during the 5 training days when animals were trained to reach the hidden platform, latency (Figure 5A) to discover the platform tends to diminish significantly in treated and non-treated rats. A three-way ANOVA for repeated measures analysis indicated significant effects of time (F (4, 50) = 933.6; *p* < 0.001), sex (F (1, 50) = 29.36; *p* < 0.0001), treatment (F (1, 50) = 12.12; *p* < 0.01), and an interaction between treatment and time (F (4, 50) = 6.408; *p* < 0.001). Yet, Post-hoc Tukey's multiple comparisons test revealed that this parameter tended to be higher in the treated male group from the third day of training, as compared to other counterparts, which suggests a learning delay induced by DEX administration at this group.

In the probe test, after removing the hidden platform to evaluate learning (Figure 5B), treated male rats showed a significant reduction preference (expressed as time passed in the target quadrant) for the target quadrant. Analysis by Two-way ANOVA indicated significant impacts of treatment (F (1, 20) = 20.43; *p* < 0.001), and sex (F (1, 20) = 12.35; *p* < 0.01). Post-hoc Tukey's multiple comparisons test revealed that male treated rats exhibited shorter latency time compared with other groups. Results obtained in this test propose that uniquely DEX treated males exhibited altered learning and spatial memory performances.

### 3.5. VEI in two-bottle choice paradigm

**Figure 6.**
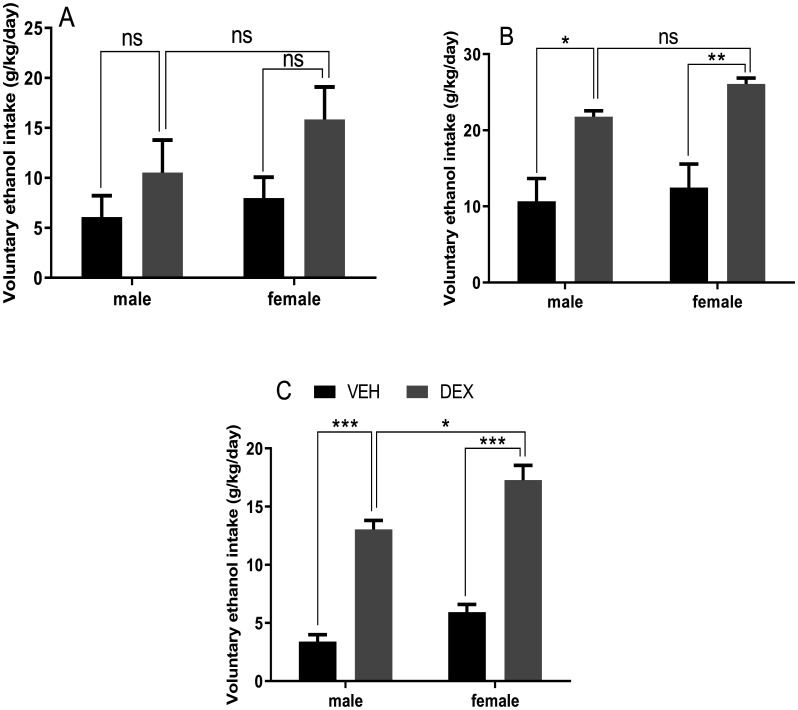
Effect of repeated dexamethasone administration on voluntary ethanol intake. Measurement of ethanol consumption according to the free-choice paradigm was carried out during three successive periods alternated by one week of withdrawal between the first (A) and second period (B) and one week of withdrawal accompanied by repeated injections of dexamethasone between the second and third period (C). Each measurement period lasted one week. During the first period of measurement consumption of ethanol was not different neither between controls and treated animals nor between males and females ((A) considered as a period of habituation). During the second period of measurement, the chronic DEX administration resulted in a greater voluntary ethanol intake without any significant difference between male and female groups (B). During the third period of measurement, the chronic DEX administration resulted in a greater voluntary ethanol intake, the treated females showed the elevated ethanol intake (B). Data represent means ± SEMs (n = 5 rats/group). VEH vehicle group, DEX dexamethasone group, ns non-significant, * *p* < 0.05, ** *p* < 0.01, *** *p* < 0.001.

Assessment of voluntary ethanol intake was carried out in three separate sessions. Each session lasted one week (Figure 6). In the first habituation session (A), ethanol intake measurement revealed no difference. In the second session (B), following one-week cessation, Chronic DEX administration resulted in a greater voluntary ethanol intake without differences between sexes. Analysis by Two-way ANOVA indicated significant impacts of treatment (F (1, 16) = 30.77; *p* < 0.0001). In the third session (C), following the second week of alcohol cessation combined with repeated daily injections of DEX, Chronic DEX administration conducted to an enhanced voluntary ethanol intake compared to the control group. Analysis by Two-way ANOVA indicated significant impacts of treatment (F (1, 16) = 148.2; *p* < 0.0001), sex (F (1, 16) = 15.42; *p* < 0.01). Post-hoc Tukey's multiple comparisons test revealed that female treated rats exhibited greater ethanol intake compared with male treated rats (*p* < 0.05).

## 4. Discussion

The aim of the present study was designed to explore the sex differences in (i) depressive-like, (ii) anxiety-like behaviors, (iii) cognitive-like performances, and (iv) voluntary ethanol intake in Wistar rats submitted to DEX-stress simulation as a depression model.

Two typical tests were performed to evaluate two main depressive-like behaviors in rats: FST to assess behavioral despair and SPT to assess anhedonia. The FST shows that chronic DEX treatment induced a depressive-like state in animals. DEX-treated rats exhibit significantly increased time of immobility. Results obtained are coherent with other works in rats and mice [29],[32],[46]. Thus, the injection of 40 mg/kg/day of Cort for 21 days, showed identical performance, [47] and [48] working on L-E rats; [49] working on Swiss Albino mice. A study conducted by [50] achieved the same results by working on ICR mice by injecting them 20 mg/kg/day of Cort for 21 day. Xu et al. [51] working on adolescent rats found similar results. These findings sustain the fact that prolonged systemic glucocorticoids administration induces despair-like behaviors. Our results also showed that females are most affected which goes in concert with some reports which indicated that male rats exhibit less immobility time than females [52],[53]. But, other studies of repeated injections of Cort increased depressive behaviors in sex-both rats, with sexual dimorphism manifested as males tended to stay more immobile than females [54]–[56].

Additionally, related to body weight evolution, rats chronically treated with DEX showed a clear decrease in body weight, this loss in weight is much observed in both depressive humans and animals [29],[32],[56],[57]. This weight reduction affected both sexes [58],[59].

In the Cort model of depression, anhedonia is a commonly evaluated behavioral indicator of a depressive-like Cort induced state. In regards to saccharin preference, our results show a significant difference between treated and non-treated rats after 21 days of DEX treatment. These findings are coherent with other works realized in rats and mice [10],[32],[60]. Our results showed a greater reduction in sucrose intake in females compared to male rats which fit with results obtained in the rat social defeat model [61]. Xu et al. [51] working on adolescent rats found similar results.

Anxiety is considered as to be one of the depression-related symptoms, it is considered as the highly comorbid behavioral complication of depression. Reports from large data showed that corticosteroid treatment has psychiatric side effects ranged from clinically significant anxiety and insomnia, to marked severe mood and psychotic disorders [62]. Taking this in mind, we asked if DEX treatment could induce anxiety-like behaviors in rats. For this goal, in the present study, evaluation of anxiety-like behaviors has been done with three test indicators: time spent in central squares of OFT arena, latency time of NSFT and time spent in open arms of EPMT.

The analysis of the performance of rats revealed a change in the behavior of groups treated with the synthetic glucocorticoid (DEX) for both sexes. This change resulted in an anxious state. In OF test, these disorders are manifested by a reduction in time spent in central cells in treated groups compared to control groups. NSF test revealed hyponeophagic behavior, which manifested an increased latency to go and chew the food pellet in DEX treated group. Finally, the EPMT test was used to assess rat neophobia, and therefore it is the best test for measuring anxiety in rodents. The anxiety-inducing effect is manifested by reduced time passed in open arms in treated groups.

In sum, there is a concordance between results obtained in this study and other findings proposing that DEX treatment induces anxiety-like behavior, Skorzewska et al. [63] indicated that in Wistar rats, treated with 20 mg/kg Cort for 25 days, time spent in OF center has been reduced. David et al. [5] showed that (35 µg/ml/day; 4 weeks, on mice) in drinking water significantly affected latency feeding time in NSFT. Studies in rats showed similar results: Cort administration (40 mg/kg; 19 days) significantly decreased time spent in open arms of EPMT [64]. A study carried out by [49] on Swiss Albino mice, showed that the injection of Cort with 40 mg/kg for 21 days resulted in marked reduced time in open arms in the EPMT indicating anxiety-like behavior. Xu et al. [51] working in adolescent rats showed that 21 DEX injections induce decreased time in open arms of the EPMT.

For sex differences, studies that take in comparison male and female rats with anxiety-like symptoms are very rare. For example, an ancient study carried out by Johnston & File [65] showed that females were more anxious than males in EPMT; the same result was obtained in the Vogel test.

Glucocorticoids can have strong peripheral effects; some effects could influence the behavioral scores like the FST immobility and the NSFT latency. One of those effects we suggest decreased locomotor activity, so we assessed if prolonged treatment with DEX affected locomotor performance in the OFT arena. In this experiment, there was no difference encountered in the DEX group compared to the non-treated group in locomotor activity expressed as distance traveled in the OFT arena. Our results are in line with other findings, Skupio et al. [32] concluded that treatment with DEX (4 mg/kg; 21 days) didn't impair locomotor activity.

Several data from the literature have shed light on stress as a strong influencer in cognitive functions like learning and memory processes [66]. Cumulative data indicate, in human studies, that hippocampal forms of memory decrease after stress and hormones stress exposure [67]. In normal individuals, treatment with elevated Cort dose decreases some kind of memory like verbal declarative form [68]. For example, alterations in declarative memory have been observed in some depressive patients with persistent hypercortisolemia or patients suffering from Cushing's pathology [69]. Also, in rodents, Anxiety/depression models have evaluated and noted cognitive impairments [70],[71]. Cognitive impairments in stressed rats under the chronic middle stress (CMS) have been noticed after behavioral testing. Thus, in MWMT spatial learning and memory was altered in CMS paradigm in mice [72], in both rats and mice this paradigm impaired recognition memory [73],[74]. Similarly, impairments in spatial memory were observed in social defeat and maternal separation [70],[75]. Rodents chronically administered by Cort show impairments in different scores related to spatial abilities [69],[67]. In fact, chronic DEX administration (200 mg 3 weeks release pellet for 9 weeks) in rats induced acquisition in radial arm maze test [76]. In another study, when Cort was used at the same dose but only for 21 days, spatial memory assessed in Barnes maze also has been impaired [77]. Sousa et al. [78] showed the same findings in spatial learning in MWMT at rats daily Cort administered (40 mg/kg) during four weeks. In our study, we used the MWMT to investigate some memory-related behaviors. Xu et al. [51] working in adolescent rats showed that 21 DEX injections induced cognition impairment in MWMT. The results obtained are in line with the mentioned findings which propose that DEX administration conducted to cognitive impairments in uniquely male rats. In this study learning and memory processes in female rats seemed to be unaffected by this stress simulation which conforms with many other studies like Buuse et al. [79] which showed that Cort-treatment had no effect on spatial memory in female rats.

Voluntary alcohol intake is facilitated by glucocorticoids. This was concluded from results in Wistar and alcohol-preferring rats with adrenalectomy which provoked diminished alcohol consumption [16], while an intracerebro-ventricular infusion of glucocorticoids enhances voluntary alcohol intake in Wistar rats [20]. In rats stressed by an early maternal separation paradigm, Peñasco et al. [45] showed that voluntary ethanol intake has been enhanced when rats were later exposed to a stressful stimulus. In our present study, results are in line with the above-mentioned data which confirms the facilitatory role of glucocorticoids on alcohol drinking.

Epidemiological data, in humans, shows that men are more prone than women related to alcohol concerns. Men seemed to have elevated rates of alcohol consumption and exhibit more dependence [80]. Conversely, surveys from animals show opposite results; male animals manifest small rates of alcohol consumption than females [81],[82]. One potential explanation, for these sex differences between humans and animals in terms of alcohol abuse, is the human sociocultural dimensions. Nevertheless, an examination of the neurobiological background may give an enhanced light to comprehend these sex discrepancies. In this study, voluntary alcohol intake measurement in two-bottle choice testing showed that female rats consumed more than males, particularly after an alcohol cessation period coupled with DEX treatment. Our results fit with a few existing preclinical data, such as [83] which reported that female adolescent rats exhibiting increased rates of social anxiety-like behavior showed an elevated alcohol intake compared to males.

## 5. Conclusion

In conclusion, DEX chronic treatment produced a range of depressive-like, (ii) anxiety-like behaviors, (iii) impaired cognitive-like performances, and (iv) increased VEI in Wistar rats submitted to DEX-stress simulation. In most of the effects induced by DEX-stress simulation used in this study, there was a difference between sexes. Females exhibited more vulnerability in DEX-induced depression. This result confirms, as in other explorations, that sex is indeed a biological variable to be taken into account either at the preclinical or clinical levels. Many other perspectives, related to the DEX model utilized in this study need to be investigated. The most important ones are the structural and molecular effects of DEX chronic treatment on the central nervous system that underlay the sex dimorphism related to DEX-induced depression.

## References

[1] Arango-Lievano M, Kaplitt MG (2015). entre la dépression et l'addiction. méd/sci.

[2] Herman JP, Cullinan WE (1997). Neurocircuitry of stress: central control of the hypothalamo–pituitary–adrenocortical axis. Trends neurosci.

[3] Tsigos C, Chrousos GP (2002). Hypothalamic–pituitary–adrenal axis, neuroendocrine factors and stress. J Psychosom Res.

[4] McEwen BS (2000). Effects of adverse experiences for brain structure and function. Biol Psychiatry.

[5] David DJ, Samuels BA, Rainer Q (2009). Neurogenesis-dependent and-independent effects of fluoxetine in an animal model of anxiety/depression. Neuron.

[6] Sapolsky RM, Krey LC, McEwen BS (1984). Glucocorticoid-sensitive hippocampal neurons are involved in terminating the adrenocortical stress response. Proc Natl Acad Sci.

[7] Sapolsky RM, Krey LC, McEWEN BS (1985). Prolonged glucocorticoid exposure reduces hippocampal neuron number: implications for aging. J Neurosci.

[8] Vyas A, Bernal S, Chattarji S (2003). Effects of chronic stress on dendritic arborization in the central and extended amygdala. Brain Res.

[9] Pham K, Nacher J, Hof PR (2003). Repeated restraint stress suppresses neurogenesis and induces biphasic PSA-NCAM expression in the adult rat dentate gyrus. Eur J Neurosci.

[10] Sterner EY, Kalynchuk LE (2010). Behavioral and neurobiological consequences of prolonged glucocorticoid exposure in rats: relevance to depression. Prog Neuro-Psychopharmacology Biol Psychiatry.

[11] Parker KJ, Schatzberg AF, Lyons DM (2003). Neuroendocrine aspects of hypercortisolism in major depression. Horm Behav.

[12] MacQueen GM, Campbell S, McEwen BS (2005). Course of illness, hippocampal function, and hippocampal volume in major depression. Focus.

[13] Alcalar N, Ozkan S, Kadioglu P (2013). Evaluation of depression, quality of life and body image in patients with Cushing's disease. Pituitary.

[14] Lamers F, van Oppen P., Comijs HC (2011). Comorbidity patterns of anxiety and depressive disorders in a large cohort study: the Netherlands Study of Depression and Anxiety (NESDA). J Clin Psychiatry.

[15] Blaine SK., Sinha R (2017). Alcohol, stress, and glucocorticoids: From risk to dependence and relapse in alcohol use disorders. Neuropharmacology.

[16] Fahlke C, Eriksson CP (2000). Effect of adrenalectomy and exposure to corticosterone on alcohol intake in alcohol-preferring and alcohol-avoiding rat lines. Alcohol Alcohol.

[17] Fahlke C, Hansen S (1996). Facilitation of ethanol consumption by intracerebroventricular infusions of corticosterone. Psychopharmacol.

[18] Piazza PV, Deroche V, Deminiere JM (1993). Corticosterone in the range of stress-induced levels possesses reinforcing properties: implications for sensation-seeking behaviors. Proc Natl Acad Sci.

[19] Brand I, Fliegel S, Spanagel R (2013). Global ethanol-induced enhancements of monoaminergic neurotransmission: a meta-analysis study. Alcohol: Clin Exp Res.

[20] Spanagel R, Noori HR, Heilig M (2014). Stress and alcohol interactions: animal studies and clinical significance. Trends Neurosci.

[21] Beery AK, Zucker I (2011). Sex bias in neuroscience and biomedical research. Neurosci Biobeha Rev.

[22] Shors TJ, Chua C, Falduto J (2001). Sex differences and opposite effects of stress on dendritic spine density in the male versus female hippocampus. J Neurosci.

[23] Cosgrove KP, Mazure CM, Staley JK (2007). Evolving knowledge of sex differences in brain structure, function, and chemistry. Biol Psychiatry.

[24] Lind KE., Gutierrez EJ, Yamamoto DJ (2017). Sex disparities in substance abuse research: Evaluating 23 years of structural neuroimaging studies. Drug Alcohol Depend.

[25] Miller LR, Marks C, Becker JB (2017). Considering sex as a biological variable in preclinical research. FASEB J.

[26] Choleris E, Galea LA, Sohrabji F (2018). Sex differences in the brain: Implications for behavioral and biomedical research. Neurosci Biobeha Rev.

[27] Clayton JA, Collins FS (2014). Policy: NIH to balance sex in cell and animal studies. Nat News.

[28] Guizzetti M, Davies DL, Egli M (2016). Sex and the lab: an alcohol-focused commentary on the NIH initiative to balance sex in cell and animal studies. Alcohol: Clin Exp Res.

[29] Sigwalt AR, Budde H, Helmich I (2011). Molecular aspects involved in swimming exercise training reducing anhedonia in a rat model of depression. Neurosci.

[30] Kenna HA, Poon AW, de los Angeles CP (2011). Psychiatric complications of treatment with corticosteroids: review with case report. Psychiatry clin Neurosci.

[31] Gaspar R, Soares-Cunha C, Domingues AV (2021). Resilience to stress and sex-specific remodeling of microglia and neuronal morphology in a rat model of anxiety and anhedonia. Neurobiol Stress.

[32] Skupio U, Tertil M, Sikora M (2015). Behavioral and molecular alterations in mice resulting from chronic treatment with dexamethasone: relevance to depression. Neurosci.

[33] Ferreira AS, Galvão S, Gaspar R, Rodrigues-Neves AC (2021). Sex-specific changes in peripheral metabolism in a model of chronic anxiety induced by prenatal stress. Eur J Clin Invest.

[34] Mesripour A, Alhimma F, Hajhashemi V (2019). The effect of vitamin B6 on dexamethasone-induced depression in mice model of despair. Nutr Neurosci.

[35] Iacono LL, Carola V (2018). The impact of adolescent stress experiences on neurobiological development. Semi cell deve biol.

[36] Yohn NL, Blendy JA (2017). Adolescent chronic unpredictable stress exposure is a sensitive window for long-term changes in adult behavior in mice. Neuropsychopharmacology.

[37] Porsolt RD, Le Pichon M, Jalfre ML (1977). Depression: a new animal model sensitive to antidepressant treatments. Nat.

[38] Abelaira HM, Réus GZ, Quevedo J (2013). Animal models as tools to study the pathophysiology of depression. Braz J Psychiatry.

[39] Hazra S, Kumar S, Saha GK (2015). Chronic Administration of Bacopa monniera Alleviates Depressive Like Behavior and Increases the Expression of ERK1/2 in Hippocampus and Pre-Frontal Cortex of Chronic Unpredictable Stress Induced Rats. Int Neuropsychiatr Dis J.

[40] Clénet F, Bouyon E, Hascoët M (2006). Light/dark cycle manipulation influences mice behaviour in the elevated plus maze. Behav Brain Res.

[41] Francis-Oliveira J, Ponte B, Barbosa APM (2013). Fluoxetine exposure during pregnancy and lactation: Effects on acute stress response and behavior in the novelty-suppressed feeding are age and gender-dependent in rats. Behav Brain Res.

[42] Wenk GL (2004). Assessment of spatial memory using the radial arm maze and Morris water maze. Curr Prot Neurosci.

[43] Monfort P, Erceg S, Piedrafita B (2007). Chronic liver failure in rats impairs glutamatergic synaptic transmission and long-term potentiation in hippocampus and learning ability. Eur J Neurosci.

[44] El Mostafi H, Elhessni A, Touil T (2020). Argan oil supplementation attenuates voluntary ethanol consumption and withdrawal syndrome promoted by adolescent intermittent ethanol in rat. Alcohol.

[45] Peñasco S, Mela V, López-Moreno JA (2015). Early maternal deprivation enhances voluntary alcohol intake induced by exposure to stressful events later in life. Neural Plast.

[46] Ruksee N, Tongjaroenbuangam W, Mahanam T (2014). Melatonin pretreatment prevented the effect of dexamethasone negative alterations on behavior and hippocampal neurogenesis in the mouse brain. J Steroid Bbiochem Mol Biol.

[47] Lebedeva KA, Caruncho HJ, Kalynchuk LE (2017). Cyclical corticosterone administration sensitizes depression-like behavior in rats. Neurosci Lett.

[48] Ali SH, Madhana RM, Athira KV (2015). Resveratrol ameliorates depressive-like behavior in repeated corticosterone-induced depression in mice. Steroids.

[49] Luo GQ, Liu L, Gao QW (2017). Mangiferin prevents corticosterone-induced behavioural deficits via alleviation of oxido-nitrosative stress and down-regulation of indoleamine 2, 3-dioxygenase (IDO) activity. Neurol Res.

[50] Bai Y, Song L, Dai G (2018). Antidepressant effects of magnolol in a mouse model of depression induced by chronic corticosterone injection. Steroids.

[51] Xu J, Wang R, Liu Y (2019). Short-and long-term alterations of FKBP5-GR and specific microRNAs in the prefrontal cortex and hippocampus of male rats induced by adolescent stress contribute to depression susceptibility. Psychoneuroendocrinology.

[52] Hill MN, Brotto LA, Lee TTY (2003). Corticosterone attenuates the antidepressant-like effects elicited by melatonin in the forced swim test in both male and female rats. Prog Neuro-Psychopharmacol Biol Psychiatry.

[53] Brotto LA, Barr AM, Gorzalka BB (2000). Sex differences in forced-swim and open-field test behaviours after chronic administration of melatonin. Eur J Pharmacol.

[54] Wintink AJ, Young NA, Davis AC (2003). Kindling-induced emotional behavior in male and female rats. Behav Neurosci.

[55] Alonso SJ, Castellano MA, Afonso D (1991). Sex differences in behavioral despair: relationships between behavioral despair and open field activity. Physiol behavi.

[56] Johnson SA, Fournier NM, Kalynchuk LE (2006). Effect of different doses of corticosterone on depression-like behavior and HPA axis responses to a novel stressor. Behav Brain Res.

[57] Feng Y, Rhodes PG, Liu H (2009). Dexamethasone induces neurodegeneration but also up-regulates vascular endothelial growth factor A in neonatal rat brains. Neurosci.

[58] Brummelte S, Pawluski JL, Galea LA (2006). High post-partum levels of corticosterone given to dams influence postnatal hippocampal cell proliferation and behavior of offspring: a model of post-partum stress and possible depression. Horm Behave.

[59] Kalynchuk LE, Gregus A, Boudreau D (2004). Corticosterone increases depression-like behavior, with some effects on predator odor-induced defensive behavior, in male and female rats. Behav Neurosci.

[60] Casarotto PC, Andreatini R (2007). Repeated paroxetine treatment reverses anhedonia induced in rats by chronic mild stress or dexamethasone. Eur Neuropsychopharmacology.

[61] Page GG, Opp MR, Kozachik SL (2016). Sex differences in sleep, anhedonia, and HPA axis activity in a rat model of chronic social defeat. Neurobiol Stress.

[62] Kenna HA, Poon AW, de los Angeles CP (2011). Psychiatric complications of treatment with corticosteroids: review with case report. Psychiatry Clin Neurosci.

[63] Skórzewska A, Bidziński A, Lehner M (2006). The effects of acute and chronic administration of corticosterone on rat behavior in two models of fear responses, plasma corticosterone concentration, and c-Fos expression in the brain structures. Pharmacol Biochem Behav.

[64] Lee B, Shim I, Lee HJ, Yang Y (2009). Effects of acupuncture on chronic corticosterone-induced depression-like behavior and expression of neuropeptide Y in the rats. Neurosci lett.

[65] Johnston AL, File SE (1991). Sex differences in animal tests of anxiety. Physiol behave.

[66] Sandi C, Pinelo-Nava MT (2007). Stress and memory: behavioral effects and neurobiological mechanisms. Neural Plast.

[67] Lupien SJ, McEwen BS (1997). The acute effects of corticosteroids on cognition: integration of animal and human model studies. Brain Res Rev.

[68] Newcomer JW, Selke G, Melson AK (1999). Decreased memory performance in healthy humans induced by stress-level cortisol treatment. Arch Gen Psychiatry.

[69] Sapolsky RM (2000). The possibility of neurotoxicity in the hippocampus in major depression: a primer on neuron death. Biol Psychiatry.

[70] Patki G, Solanki N, Atrooz F (2013). Depression, anxiety-like behavior and memory impairment are associated with increased oxidative stress and inflammation in a rat model of social stress. Brain Res.

[71] Richter SH, Zeuch B, Lankisch K (2013). Where have I been? Where should I go? Spatial working memory on a radial arm maze in a rat model of depression. PloS One.

[72] Song L, Che W, Min-Wei W (2006). Impairment of the spatial learning and memory induced by learned helplessness and chronic mild stress. Pharmacol Biochem Behav.

[73] Orsetti M, Colella L, Dellarole A (2007). Modification of spatial recognition memory and object discrimination after chronic administration of haloperidol, amitriptyline, sodium valproate or olanzapine in normal and anhedonic rats. Int J Neuropsychopharmacology.

[74] Elizalde N, Gil-Bea FJ, Ramirez MJ (2008). Long-lasting behavioral effects and recognition memory deficit induced by chronic mild stress in mice: effect of antidepressant treatment. Psychopharmacology.

[75] Couto FS, Batalha VL, Valadas JS (2012). Escitalopram improves memory deficits induced by maternal separation in the rat. Eur J Pharmacol.

[76] Dachir S, Kadar T, Robinzon B (1993). Cognitive deficits induced in young rats by long-term corticosterone administration. Behav Neural Biol.

[77] Coburn-Litvak PS, Pothakos K, Tata DA (2003). Chronic administration of CORT impairs spatial reference memory before spatial working memory in rats. Neurobiol Learn Mem.

[78] Sousa N, Lukoyanov NV, Madeira MD (2000). Reorganization of the morphology of hippocampal neurites and synapses after stress-induced damage correlates with behavioral improvement. Neurosci.

[79] van den Buuse M, Buret L, Hill R (2020). Involvement of brain-derived neurotrophic factor (BDNF) in the long-term memory effects of glucocorticoid stimulation during adolescence/young adulthood. Behav Brain Res.

[80] Spanagel R, Durstewitz D, Hansson A (2013). A systems medicine research approach for studying alcohol addiction. Addict Bbiol.

[81] Frank J, Cichon S, Treutlein J (2012). Genome-wide significant association between alcohol dependence and a variant in the ADH gene cluster. Addict Biol.

[82] Biernacka JM, Geske JR, Schneekloth TD (2013). Replication of genome wide association studies of alcohol dependence: support for association with variation in ADH1C. Plos One.

[83] Varlinskaya EI, Truxell EM, Spear LP (2015). Sex differences in sensitivity to the social consequences of acute ethanol and social drinking during adolescence. Behav Brain Res.

